# Telomeres, NAFLD and Chronic Liver Disease

**DOI:** 10.3390/ijms17030383

**Published:** 2016-03-15

**Authors:** Benedetta Donati, Luca Valenti

**Affiliations:** Department of Pathophysiology and Transplantation, Università degli Studi di Milano, Fondazione IRCCS Ca’ Granda Ospedale Policlinico Milano, 20122 Milano, Italy; benedetta.donati@unimi.it

**Keywords:** telomere, telomerase, liver disease progression, nonalcoholic fatty liver disease, cirrhosis, hepatocellular carcinoma

## Abstract

Telomeres consist of repeat DNA sequences located at the terminal portion of chromosomes that shorten during mitosis, protecting the tips of chromosomes. During chronic degenerative conditions associated with high cell replication rate, progressive telomere attrition is accentuated, favoring senescence and genomic instability. Several lines of evidence suggest that this process is involved in liver disease progression: (a) telomere shortening and alterations in the expression of proteins protecting the telomere are associated with cirrhosis and hepatocellular carcinoma; (b) advanced liver damage is a feature of a spectrum of genetic diseases impairing telomere function, and inactivating germline mutations in the telomerase complex (including *human Telomerase Reverse Transcriptase* (*hTERT)* and *human Telomerase RNA Component (hTERC)*) are enriched in cirrhotic patients independently of the etiology; and (c) experimental models suggest that telomerase protects from liver fibrosis progression. Conversely, reactivation of telomerase occurs during hepatocarcinogenesis, allowing the immortalization of the neoplastic clone. The role of telomere attrition may be particularly relevant in the progression of nonalcoholic fatty liver, an emerging cause of advanced liver disease. Modulation of telomerase or shelterins may be exploited to prevent liver disease progression, and to define specific treatments for different stages of liver disease.

## 1. Introduction

**Figure 1:**
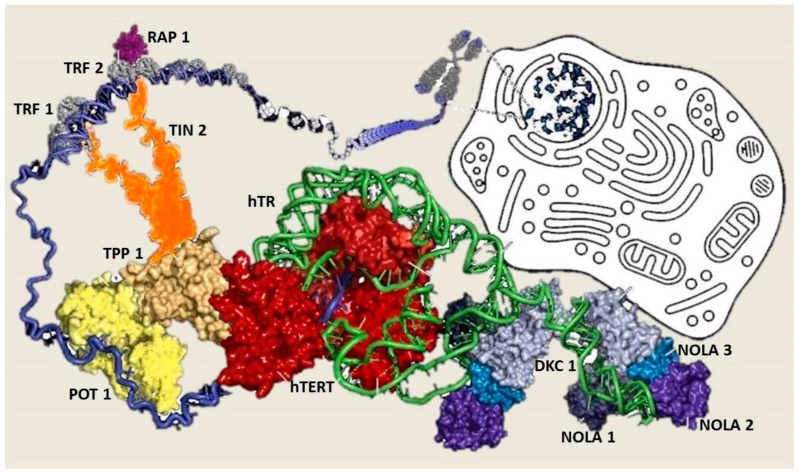
Model representing the telomeres associated proteins. Telomerase (including hTERT (**red**) and hTERC (**green**)) represents the principal catalytic subunit. The Shelterin complex is anchored by binding of the proteins TRF1 and TRF2 to double-stranded telomeric repeats. TRF1 and TRF2 are bridged to the single-stranded telomeric-repeat G-strand DNA-binding protein POT1 through TIN2 and TPP1. Additionally, shelterin RAP1 binds directly to TRF2. Dyskerin complex involving NOLA proteins, interacts and stabilizes the non-overlapping regions of hTERC.

In humans, telomeres consist of thousands copies of six base repeats (TTAGGG) located at the extremities of the chromosomes that protect chromosomes tips from end-to-end fusion, rearrangement and translocation. Telomere length is progressively shortened at each mitosis, due to the inability of the DNA polymerase complex to replicate the very 5′ end of the lagging strand (attrition). For this reason, telomere shortening may function as a “mitotic clock” to sense somatic cells aging. When telomeres become critically short, a DNA-damage program is activated, leading to apoptosis or cell senescence. On the contrary, immortal cells (cancer, stem and germ cells) constitutionally express telomerase, a ribonuclear enzymatic complex associated with telomeres that is responsible for stabilizing telomere length by synthesizing new DNA sequences and adding them to the end of the chromosomes during DNA replication [1]. Telomerase comprises two essential components: Telomerase reverse transcriptase (hTERT) and its RNA template, the telomerase RNA component (hTERC). Dyskerin complex binds to hTERC, in order to protect it and to stabilize the telomerase complex. It includes four nucleolar proteins: Dyskerin (DKC1) and Nucleolar protein family A member 1, 2 and 3 (NOLA1-NOLA2-NOLA3) [2,3,4]. Besides telomerase, the Shelterin complex, which binds specifically to telomeres, plays a fundamental role in the protection of chromosome ends facilitating telomerase-based telomere elongation [5]. It is composed of six core proteins: the telomeric repeat binding factors 1 and 2 (TRF1-TRF2) that bind telomeric double strand DNA, the protection of telomeres 1 (POT1)*,* which binds the 3′ telomeric region of single strand DNA avoiding the degradation by nuclease, and the TRF-1 interacting protein 2 (TIN2), the POT1-TIN2 organizing protein (TPP1) and the repressor/activator protein 1 (RAP1), that interact with the other proteins bound to telomere stabilizing the complex (Figure 1; [6,7]). Mutations of proteins involved in maintenance and repair of telomeres are responsible for telomeropathies [8,9]: a spectrum of progressive genetic diseases exemplified in the most severe cases by dyskeratosis congenita (DKC), whose common autosomal recessive form is caused by mutations in *DKC1*. They are degenerative and age-dependent diseases, characterized by premature senescence of the stem cell compartment, determining increased risk of organ failure and cancer, with possible involvement of the hematopoietic compartment, lungs, mucous membranes, skin, and also the liver. Consistently, loss-of-function mutations in *hTERT* and *hTERC* may cause a spectrum of familial liver diseases [10]. Telomere length is a strong hereditable tract and telomere shortening is accentuated in chronic degenerative condition associated with high cell replication rate. Thus, involvement of telomeres and telomerase mutations seems to be important in predisposition to liver disease progression towards hepatocellular carcinoma (HCC). Indeed, the incidence of HCC increases with age, and, in particular, in nonalcoholic fatty liver disease (NAFLD), where there is a strong aggregation of familial cases [11].

## 2. Telomerase and Telomere Diseases

### 2.1. Telomere Shortening Related to Cellular Senescence Characterizes Human Cirrhosis

The role of ageing in liver fibrosis progression has been largely demonstrated, and older age and duration of liver disease remain the major and more validated risk factors for liver disease progression, together with male gender and alcohol abuse [12,13]. Cellular ageing is generally referred to as replicative senescence, a condition strictly linked to telomerase and telomere biology. Indeed, telomere shortening limited the replicative capacity of cells and the number of cells participating in tissue regeneration. Thus, the regenerative potential of an organ depends on the size of the population of cells with sufficient telomere reserves required for cell proliferation. Consistently, in chronic disease associated with tissue regeneration, such as cirrhosis, an elevated regenerative pressure is generated on the proliferating subpopulation of cells, which undergoes several rounds of cell division that, in turn, accelerate the rate of telomere shortening [14]. When telomeres become critically short, a DNA damage program is activated, leading to cell senescence or apoptosis (due to the Hayflick limit), further reducing the number of cell with regenerative capacity. 

Several lines of evidence correlate shortened telomeres with liver fibrosis. Kitada *et al.* [15] first described a progressive reduction of telomere length during liver disease progression. Urabe *et al.* [16] confirmed these data and described telomerase re-activation in poorly differentiated HCC, consistently with an increase of telomere length compared to those well differentiated. In the normal liver, progressive telomere shortening has been correlated with age. Consistently, reduction of telomere length in cirrhotic tissue was more marked in patients who developed cirrhosis at younger age [17]. Additionally, reduction of telomere length is considered a hallmark of cirrhotic tissue independently of the etiology of liver disease (e.g., viral hepatitis, autoimmune hepatitis, alcohol abuse…) [18]. Thus, excessive telomere shortening, caused either by telomerase gene mutations or acquired factors, may impair the hepatocyte regenerative ability in response to chronic injury, facilitating fibrosis progression [19,20]. A causal role of telomere shortening in fibrosis progression has been experimentally demonstrated in telomerase deficient mice. After three generations, these mice developed shortened telomeres and displayed diminished capacity for liver regeneration, and with accelerated development of cirrhosis after liver injury. On the contrary, overexpression of TERT activity improved liver function and protected mice from development of hepatic steatosis and fibrosis [21].

Consistently, shortened telomere length in cirrhotic patients was correlated with the expression of known markers of cellular senescence, such as β-galactosidase, p16, p21 and p53 not only in hepatocytes but also in non-parenchymal cells, such as biliary cells [22,23]. The p53 protein represents the key regulator point for various signaling pathways of senescence: p53 phosphorylation and consequent activation inhibits cell division primarily inducing p21 expression, which, in turn, activates pRb through inhibition of a cyclin-dependent kinase (Cdk) complex. The activated pRb inhibits the transcription of E2F target genes that are required for cell cycle progression. pRB can alternatively be activated by p16, another Cdk inhibitor, that typically accumulates in senescent cells [23].

Cellular senescence may have a dual role in liver disease: in a first phase, it seems to contribute to liver impairment by reducing the hepatocytes and progenitor cell population, while, in a second phase, the subsequent senescence of HSC (epatic stellate cells) due to long-standing activation of fibrogenesis may protect from further fibrosis progression [24,25,26]. In particular, progression of human fibrosis is often characterized by a state of chronic inflammation that results in a condition of cell death and tissue regeneration, involving also a massive expansion of hepatic progenitor cells in order to restore the lost hepatocytes. Ductular reaction typical of this condition has been shown to produce chemotactic stimuli for induction of inflammatory cells and activation of pro-fibrotic hepatic stellate cells (HSC). Moreover, due to the epithelial to mesenchymal transition, progenitors and biliary epithelial cells may provide a portion of myofibroblasts, contributing to fibrosis progression [27]. When the wound is filled, the activated HSC undergo apoptosis or cellular senescence and consequently are eliminated by immune cells. In this way, HSC induce the recruitment of other immune cells at the site of tissue injury that, in turn, help in arresting liver fibrosis progression. However, it has recently been shown that later, senescent HSC may favor HCC development by secreting pro-carcinogenic mediators (the senescence associated secretory program: SASP) [28].

### 2.2. Telomerase Mutations Are Hallmarks of Liver Fibrosis

Genetic studies have proven that mutations in telomerase represent the underlying cause of accelerated telomere shortening and organ failure in some rare human diseases, including some forms of DKC [29], which may be characterized by liver injury and development of complications of portal hypertension. Moreover, evidence suggests that telomere attrition is also involved in liver disease progression in humans. Indeed, a spectrum of familial liver disease with autosomal dominant transmission and incomplete penetrance has been associated with inheritance of *hTERT* and *hTERC* mutations [10,30]. In these pedigrees, liver disease was characterized by development of steatosis, with possible progression to cirrhosis and HCC. Furthermore, a significant enrichment of missense mutations in the *hTERT* and *hTERC* genes was observed in 7% of patients and one patient, respectively, of a US cohort including 134 patients with cirrhosis of different etiologies (NAFLD, but also alcohol abuse and Hepatitis C virus infection), as compared to healthy controls. These mutations impaired hTERT enzymatic activity, as they were associated with reduced telomere length in the peripheral blood of patients and reduced telomerase activity *in vitro* [19]. These data were substantially confirmed in a larger series of 521 German patients with cirrhosis, of whom 3% carried functional *hTERT* mutations again independently from the etiology of the liver disease [31]. These observations indicate that, in at least a proportion of patients who developed cirrhosis, fibrosis progression may be favored by genetic risk variants facilitating telomere shortening and cell senescence in the presence of triggering factors.

### 2.3. Telomere Shortening Induces Genomic Instability in Hepatocellular Carcinoma (HCC)

Thus, telomere shortening is a hallmark of cirrhosis, the main risk factor for the development of liver cancer [32]. The state of chronic inflammation characteristic of injured liver, results *per se* in oxidative DNA damage leading to genomic and epigenomic alterations, pushing cells toward a malignant phenotype. Deregulation of key oncogenes and tumor-suppressor genes, such as *TP53*, *β-catenin, ErbB* *receptor family members* and *p16(INK4a)* have been observed both in early and advance HCC. Impaired function of p53 most likely induces alterations in DNA damage response machinery, resulting in loss of DNA repairing and avoiding cellular apoptosis, thus contributing to an increased mutation rate. Moreover, aberrant DNA methylation patterns have been reported in the earliest stages of hepatocarcinogenesis, and to a greater extent in tumor progression. Finally, karyotypic analysis of HCCs revealed that recurrent regions of copy number change and allelic imbalances are present in 90% of cases, thus highlighting the possibility for new cancer gene targets reside in these loci [33,34]. In this context, telomere shortening may favor carcinogenesis by directly facilitating genomic instability. Telomere shortening plays a pivotal role in inducing genomic alteration first favoring chromosomes segregation defect. Indeed, shortened telomeres have been associated with the typical karyotipic alterations in HCC (chromosome 8 alterations), especially in the presence of *TP53* mutations [33,35].

Moreover, loss of *hTERT* has been shown to affect the overall configuration of chromatin and to diminish the capacity for DNA repair of double strand breaks (DSB) [36]. Therefore, current data suggest a model whereby telomere shortening drives chromosomal instability during early stages of hepatic carcinogenesis, while telomerase re-activation is involved in malignant progression, as it restores chromosomal stability necessary for cellular immortalization.

### 2.4. Elongation of Telomeres and Telomerase Complex Reactivation during Advance Hepatocarcinogenesis

While the majority of tumors display shortened telomeres compared to non-neoplastic tissues, nevertheless telomere lengthening has been observed in various tumors at advanced stage, including colorectal, and head and neck cancers [37]. In HCC tissues, long telomeres and increased telomerase activity were also shown to be a significant reflection of poor prognostic factors, associated with clinicopathological features of aggressive behavior [38]. Indeed, HCC tumor progression is associated with the reactivation of telomerase, which is necessary for the immortalization of the neoplastic clone [39,40]. Accordingly, *hTERT* was found upregulated in dysplastic liver nodules and to be more than 10-fold induced in overt HCC tissue compared to the surrounding non-neoplastic tissue [41] independently from the etiology of liver disease [42].

On the contrary, a specific gene signature of the Shelterin complex has been identified for each cause of liver disease. Indeed, Shelterin overexpressed in HCC developed upon HCV infection or in the presence of alcohol abuse, and displayed a diminished expression in HCC developed upon HBV infection [5]. In particular, longer telomeres have been observed in HCCs expressing markers of stemness, such as CK19, EpCAM and CD133, generally considered more aggressive than the conventional, negative for these markers [43,44]. It is known that there is heterogeneity in the expression patterns of stemness-related markers within the same tumors. Interestingly, the analysis of telomere length among different cells according to EpCAM expression status has shown that longer telomeres were present in HCC tumor cells that expressed EpCAM, compared to tumor cells that were EpCAM-negative [45]. Additionally, stemness–related markers were correlated with the expression of the Shelterin proteins. Increased TPP1, TRF2, RAP1, and POT1 expression were observed in HCC tissues expressing ‘‘stemness’’-related markers compared to conventional HCCs, and their expression was correlated with poorer prognosis and reduced disease-free survival [45]. On the other hand, shortened telomeres and low POT1 expression have been observed in HCCs expressing HepPar1, a marker of hepatocytes differentiation. Additionally, Kim *et al.* [46] demonstrated that TPP1 expression was correlated with hTERT expression, supporting previous findings indicating TPP1 as a positive regulator of telomere maintenance that may represent a good target for cancer therapy as it plays a dominant role in the recruitment of hTERT to telomeres.

Elongation of telomere may also be due to higher expression of DKC1 in HCC compared to noncancerous liver tissue where the level of the protein was absent or very low. DKC1 expression has been validated as an independent risk factor for adverse overall mortality, and it was correlated with advanced HCC clinical stage (grade III–IV) and recurrence independently of hTERT expression [47]. Considering that *DKC1* is the direct and conserved transcriptional target of c-myc responsible for proliferative activity of cancer cells [48], this suggests that the role of DKC1 on cancer progression may be independent of its involvement in telomerase complex function.

Additionally, elongation of telomeres in 7% of HCC cases is associated with alternative lengthening of telomeres (ALT), the telomerase-independent telomere maintenance mechanism, which is thought to be dependent on homologous recombination. The ALT-positive cells are characterized by telomere length heterogeneity, as well as increased chromosomal instability [49].

### 2.5. Mechanisms of Reactivation of Telomerase in HCC Tissue

Several mechanisms have been shown to lead to telomerase activation during hepatic carcinogenesis. *hTERT* promoter mutations have been described as the most frequent somatic genetic alteration in HCC, with an overall frequency of 60% in Western countries, in particular in patients with chronic HCV infection [50,51]. Interestingly, these somatic mutations occur not only in cancer tissue but in 6%–19% of the cases have been observed also in the early cirrhotic tissue, while usually somatic mutations in oncogene or oncosuppressor genes occur in a more advanced stage of tumorigenesis [51,52]. These promoter mutations represent the most important mechanism of reactivation of telomerase during hepatocarcinogenesis. Indeed, they create new binding sites for specific transcription factors, which consequently induce hTERT overexpression [53,54]. No promoter mutations have been individuated in studies involving cholangiocarcinoma [52] and hepatoblastoma [55], while a minority of patients affected by hepatocholangiocarcinoma presented these kinds of mutations. This evidence suggests that telomerase involvement is dependent on the origin of the cancer cells [56]. In HCC, due to HBV infection, the reactivation of telomerase is generally due to the insertion of the HBV virus in *hTERT* gene, more frequently in the promoter [57,58]. Integration of HBV was detected in 22% of the HBV positive samples, whereas *hTERT* focal amplification, another mechanism likely inducing increased telomerase activity, in 6.7% of the cases. In the same study, *hTERT* promoter mutations were mutually exclusive with HBV genome integration in the *hTERT* locus and were almost mutually exclusive with *hTERT* focal amplifications [59].

### 2.6. Telomerase Promotes Hepatic Carcinogenesis by Multiple Pathways

Besides telomere protection and maintenance, several *in vitro* and *in vivo* studies in which *hTERT* has been exogenously expressed revealed novel telomerase functions in tumorigenesis independently of *hTERC* [60]. First, hTERT can act as a transcription factor in the Wnt-β-catenin signaling pathway, regulating the expression of Wnt target genes, which play a role in tumorigenesis. Indeed, hTERT interacts with BRG1, a chromatin remodeler binding to β-catenin and involved in the Wnt signaling [61], and promotes the expression of several β-catenin target genes in a BRG1-dependent way. Consistently, *hTERT* was found to interact with the same promoter elements recognized by BRG1 and β-catenin [62]. Actually, the relationship between hTERT and the Wnt-β-catenin pathway is bidirectional: indeed, *β-catenin* deficient human cell lines showed shorter telomere and reduced telomerase activity, and *hTERT* appears as a direct target of β-catenin through the binding to TCF4 transcription factor [63]. Furthermore, hTERT and BRG1 interact with nucleostemin, a GTP-binding protein overexpressed in stem cells and cancers [64], which is essential to drive transcriptional programs relevant for the maintenance of the cancer stem cells phenotype [65]. In this case, hTERT contributes to tumorigenesis increasing the proportion of stem cells within a tumor.

Further functions of hTERT in tumorigenesis are related to its localization in mitochondria. Here, telomerase plays a role as an RNA-dependent RNA polymerase (RdRP) paired to a mitochondrial non-coding RNA, the mitochondrial RNA processing endoribonuclease (RMRP) [66]. hTERT represents the only RdRP identified in mammals and hTERT-RMRP complex leads to the production of double-stranded RMRP RNA molecules, subsequently processed into 22-nucleotide siRNAs by RNA-induced silencing complex (RISC) [66]. Since RMRP has several cellular functions, including mRNA cleavage of cell cycle genes [67], hTERT may influence cellular proliferation, both increasing cell division and reducing apoptosis, independently of activation of Wnt signaling.

Finally, hTERT can increase cancer cell fitness, improving mitochondrial activity and resistance to apoptosis. Indeed, mt-TERT, through its reverse transcriptase domain, can provide mt-DNA replication and repair using mt-tRNAs as the template [68]. Additionally, Sahin *et al.* [60,69] noticed that *Tert* and *Terc* late generation knockout mice showed a p53-mediated repression of peroxisome proliferator-activated receptor gγ coactivator-1 α and β (Pgc-1α and Pgc-1β), the master regulators of mitochondrial physiology and metabolism, resulting in altered mitochondrial biogenesis and function and increased reactive oxygen species.

### 2.7. Telomeres and Nonalcoholic Fatty Liver Disease (NAFLD)

Following the epidemic of obesity and type 2 diabetes, NAFLD is becoming the most frequent liver disease in Western countries. Established risk factors for disease progression in NAFLD include older age and presence of features of the metabolic syndrome, such as obesity, insulin resistance, and hypertension. However, progression of liver disease to cirrhosis and HCC is generally limited to the subgroup of patients who developed non-alcoholic steatohepatitis (NASH), a condition characterized by active inflammation and fibrosis [70]. Genetic factors have also been shown to influence disease progression in NAFLD. Besides the most validated factors influencing lipid metabolism, such as the I148M variant of *PNPLA3*, the influence of variants involved in fibrogenesis has recently been described.

Genetic data indicate that NAFLD is commonly observed in patients with telomeropathies, suggesting that steatosis may either be a consequence of hepatocellular senescence, as also observed in animal models, or a trigger for liver disease progression [10,21]. Fibrosis stage and liver disease progression are also strictly linked to cell senescence. Consistently, hepatocyte expression of p21, playing a pivotal role in the induction and maintenance of cellular senescence, was associated with fibrosis stage in NAFLD and increase liver related morbidity and mortality [71]. Additionally, the rs762623 variant in the promoter region of *Cyclin-dependent Kinase 1A* (*CDKN1A*) gene, encoding for p21 protein, was associated with the development but not the progression of fibrosis in NAFLD independently from well-recognized *PNPLA3* I148M status [72]. This polymorphism has been associated with reduced p21 expression by abolishing an E2F transcription factor binding site. Thereby these data suggest that *CDKN1A* rs762623 G > A polymorphism favors HSC proliferation by limiting p21 induction, due to DNA damage and telomere shortening, but it may not predispose to severe fibrosis because it antagonizes cellular senescence [73]. Interestingly, *CDKN1A* variants have previously been described in association with rapid progression of idiopathic pulmonary fibrosis, another degenerative condition characterized by cellular senescence and impairment of telomeres [74].

Telomere attrition may also be involved in mediating cancer susceptibility in NAFLD. We reported the occurrence of HCC in NAFLD in a family where a novel missense *hTERT* mutation was segregated with idiopathic familial pulmonary fibrosis and NAFLD. This rare Glu668Asp variant located in the motif 3c of the reverse transcriptase domain of the protein likely led to reduced telomeres length by directly interfering with hTERT enzymatic activity [75]. This finding suggested us to investigate the presence of *hTERT* germline coding mutations in a cohort of patients who developed HCC without recognized risk factors (cryptogenic) or were affected only by NAFLD, which, in the absence of other predisposing conditions, is *per se* a relatively weak risk factor for progressive liver disease. We observed a highly significant enrichment of germline coding mutations in NAFLD HCC. In fact, 10% of NASH HCC were carriers of mutations, while no mutations were identified in 30 NASH cirrhosis and in healthy controls. The rare mutations modifying the sequence of the protein identified (three missense and one frameshift) were located in the N-terminal domain of interaction with hTERC or in the catalytic domain, likely impairing the activity of the telomerase complex. However, the relatively small number of patients analyzed did not allow for correlation of the presence of *hTERT* mutations with HCC prognosis. Additionally, in the same study, we found that telomeres are progressively shortened in peripheral blood leukocytes of NAFLD HCC patients compared to cirrhosis and controls [76]. These data point out a possible causal role for telomere attrition and telomerase mutations in influencing susceptibility towards HCC in NAFLD patients. As telomere shortening was not always correlated with the presence of *hTERT* mutations, this suggests that mutations in other genes contributing to the maintenance of telomeres or epigenetic mechanisms may result in a similar phenotype (genetic heterogeneity) and contribute to the phenotypic expression of heterozygous *hTERT* mutations.

## 3. Conclusions

**Figure 2:**
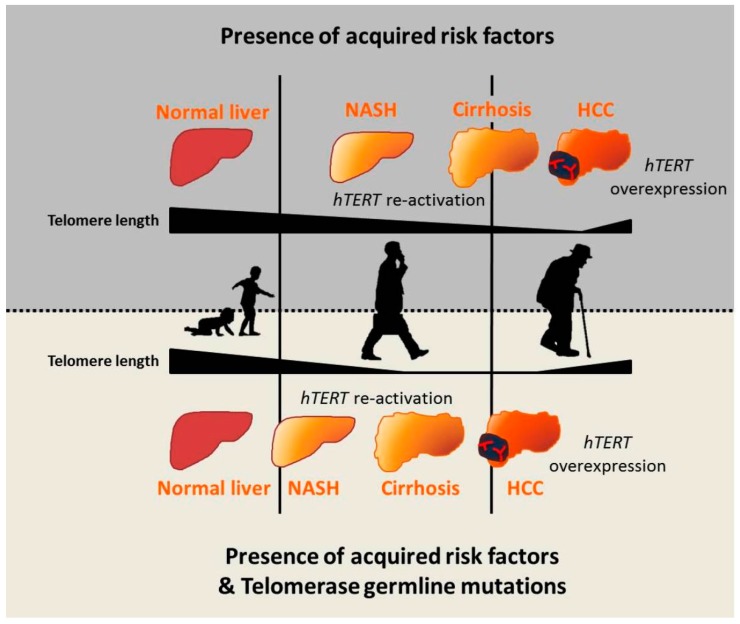
Hypothesis for telomeres’ role in pathogenesis of nonalcoholic fatty liver disease (NAFLD) progression toward cirrhosis and hepatocellular carcinoma (HCC). The model shows that, in the presence of triggering acquired risk factors such as obesity and insulin resistance, the liver undergoes cycle of damage and regeneration that requires telomerase re-activation. However, degenerative chronic conditions lead to telomere shortening and fibrosis progression towards cirrhosis, the main risk factor for HCC. In carriers of telomerase germline loss-of-function mutations, this process is accelerated due to telomerase inability to elongate telomeres, thus impairing tissue regeneration. Moreover, telomerase mutations may have a direct pro-carcinogenic effect by inducing genomic instability. Finally, telomere re-elongation in cancer tissue was triggered by different mechanisms, among which, overexpression of *hTERT* is necessary for the immortalization of the neoplastic clone.

Telomeres and telomerase play an important role in the onset and progression of liver disease independently of the underlying etiology. However, the role of telomere attrition and cell senescence is most likely magnified in NAFLD, where genetic risk factors and ageing have a large impact on the predisposition to advanced liver damage in combination with acquired risk factors. The role of telomeres in the pathogenesis of liver disease may be explained by the following hypothesis. Triggering factors, such as obesity and insulin resistance in the case of NAFLD, induce a condition of chronic hepatic damage and regeneration characterized by progressive hepatocytes telomere shortening and senescence. When hepatocytes reach senescence, liver regeneration decreases, but chronic damage remains. Concomitantly, other cell types, such as HSCs, become activated and form fibrotic tissue in area of hepatocyte loss. In this context, germline *hTERT* loss-of-function mutations accelerate telomere shortening, favoring fibrosis development and thus creating a favorable microenvironment for cancer onset. Moreover, telomere attrition and germline *hTERT* loss-of-function mutations may exert a direct pro-carcinogenic effect by promoting genomic instability, both inducing telomere shortening and impairing telomerase activity in DNA repair and chromatin organization [36]. Within this context, the presence of heterozygous mutations does not prevent the reactivation of the telomerase wild type allele at later stages of carcinogenesis, which is necessary for the indefinite replication of the neoplastic clone (Figure 2).

Several studies suggest the use of telomerase inhibitors for HCC treatment. These molecules will hopefully be able to arrest early tumor growth by blocking telomerase, having an almost immediate effect since they likely act on a phenotype of still short telomeres [77]. Moreover, they could arrest inflammatory and HSC telomerase activity, and, consequently, telomere elongation, which has been described as a feature of cirrhotic tissue surrounding tumors [18], thus having a beneficial effect both on the cirrhotic and the cancer tissue. Additionally, inhibition of telomerase may enhance chemosensitivity of cancer cells to chemotherapeutic agents [78]. *Vice versa*, treatment based on molecules that activate telomerase may be useful at the first stage of liver disease and in patients carrying telomerase complex mutations, in order to permit tissue regeneration by avoiding hepatocyte telomere shortening and senescence. This could be exploited by transplantation of liver cells engineered for *hTERT* gene expression, by directly delivering hTERT to the organ, or by small molecules enhancing telomerase activity. However, to date, it is not known how to manage both the carcinogenic potential of *hTERT*-immortalized hepatocytes, and the hepatotoxicity linked to gene delivery [77].

Interestingly, both the inhibition and the activation of telomerase may be useful in different stages of liver disease, and, at the same time, may have important side effects due also to the impairment of the physiological expression of this gene in other organs and tissues. Thus, how to act in order to modulate telomerase activity remains controversial. Further studies are necessary in order to better understand the impact of telomeres and telomerase on the different levels of liver disease progression, and consequently how to act to prevent telomerase related damage.

## Abbreviations

The following abbreviations are used in this manuscript:

NAFLD	Non-alcoholic fatty liver disease
HCC	Hepatocellular carcinoma
*hTERT*	Human telomerase Reverse Transcriptase
*hTERC*	Human telomerase RNA Component
*DKC1*	Dyskerin
*NOLA1-NOLA2- NOLA3*	Nucleolar protein family A member 1, 2 and 3
*TRF1-TRF2*	Telomeric repeat- binding factors 1 and 2
*POT1*	Protection of telomeres 1
*TIN2*	TRF-1 interacting protein 2
*TPP1*	POT1-TIN2 organizing protein
*RAP1*	repressor/activator protein 1
*pRb*	Retinoblastoma 1
*Cdk*	Cyclin-dependent kinase
*E2F*	Transcription factor E2F
*ErbB*	Epidermal Growth Factor Receptor family members
*INK4a*	Cyclin-dependent kinase inhibitor 2A
*K19*	Keratin 19
*EpCAM*	Epithelial Cell Adhesion Molecule
ALT	Alternative Lengthening of Telomeres
*BRG1*	SWI/SNF related, matrix associated, actin dependent regulator of chromatin, subfamily a, member 4
*TCF4*	Transcription factor 4
*RdRP*	RNA-dependent RNA polymerase
*RMRP*	RNA component of mitochondrial RNA processing endoribonuclease
siRNA	Silencing RNA
RISC	RNA-induced silencing complex
*Pgc-1α/β*	Peroxisome proliferator-activated receptor γ coactivator-1 α/β
NASH	Non-alcoholic steatohepatitis
*CDKN1A*	Cyclin-dependent kinase inhibitor 1A
